# Microbiological and Chemical Characteristics of Wet Coffee Fermentation Inoculated With *Hansinaspora uvarum* and *Pichia kudriavzevii* and Their Impact on Coffee Sensory Quality

**DOI:** 10.3389/fmicb.2021.713969

**Published:** 2021-08-04

**Authors:** Hosam Elhalis, Julian Cox, Damian Frank, Jian Zhao

**Affiliations:** ^1^Food Science and Technology, School of Chemical Engineering, The University of New South Wales, Sydney, NSW, Australia; ^2^Commonwealth Scientific Industry Research Organisation (CSIRO), North Ryde, NSW, Australia

**Keywords:** *Hansinaspora uvarum*, *Pichia kudriavzevii*, yeasts, coffee fermentation, volatiles

## Abstract

*Hansinaspora uvarum* and *Pichia kudriavzevii* were used as starter cultures to conduct inoculated wet fermentations of coffee beans, and their growth, metabolic activities and impact on the flavor, aroma and overall sensory quality of coffee were compared with spontaneous fermentation (control). *H. uvarum* and *P. kudriavzevii* dominated the fermentations, growing to maximum populations of about 10.0 log CFU/ml compared with 8.0 log CFU/ml in the spontaneous fermentation. The dominance of the inoculated yeasts led to faster and more complete utilization of sugars in the mucilage, with resultant production of 2–3 fold higher concentrations of metabolites such as glycerol, alcohols, aldehydes, esters, and organic acids in the fermented green beans. Cup tests showed coffee produced from the inoculated fermentations, especially with *P. kudriavzevii*, received higher scores for flavor, aroma and acidity than the control. The findings of this study confirmed the crucial role of yeasts in the wet fermentation of coffee beans and their contribution to high quality coffee, and demonstrated the potential *H. uvarum* and *P. kudriavzevii* as starter cultures in the process.

## Introduction

The wet process is one of the major primary processing methods for coffee beans and able to produce coffee with high qualities (Mussatto et al., 2011; Brando and Brando, 2014). In the process de-pulped ripe coffee cherries are subjected to underwater fermentation for 6–72 h, and dried (Schwan and Wheals, 2004). The fermentation is performed mainly to remove the mucilage layers still stuck to the coffee beans (Brando and Brando, 2014). Furthermore, it was reported that the process improves coffee flavor and aroma by producing desirable microbial metabolites such as higher alcohols, esters, aldehydes, and organic acids (Mussatto et al., 2011; Elhalis et al., 2020a). The wet fermentation is largely conducted in a traditional, uncontrolled manner where indigenous microflora grow spontaneously, leading to a complex microbial ecology (Avallone et al., 2001; Silva et al., 2008). Conducting the fermentation in this way increases the probability of producing products with inconsistent and unpredictable quality (Durso and Hutkins, 2003). Transforming the fermentation into a more efficient and controllable industrial process can be achieved by using defined starter cultures. Wild type endogenous isolates originated from the natural ecosystem of wet fermentation can be a good source to search for promising candidates of functional starter cultures (Leroy et al., 2006; Wouters et al., 2013; Pereira et al., 2015).

A wide range of microorganisms have been isolated from coffee fermentations, including yeasts, filamentous fungi, lactic acid bacteria, acetic acid bacteria, *Enterobacteriaceae*, and *Bacillus* spp. (Avallone et al., 2001; Djossou et al., 2011; Elhalis et al., 2020a). Yeasts are one of the frequent isolates from coffee fermentation in multiple locations over the globe with *Hansinaspora uvarum*, *Debaryomyces hansenii*, *Pichia kluyveri*, *P*. *anomala*, *Saccharomyces cerevisiae*, *Candida*, and *Torulaspora delbrueckii* being the common species (Masoud et al., 2004; Silva et al., 2008; Vilela et al., 2010; Pereira et al., 2014; Elhalis et al., 2020a). Several yeast species isolated from spontaneous coffee fermentations have been reported to possess mucilage degradation capability, produce flavoring compounds, and suppress the growth of mycotoxin producing filamentous fungi (Masoud et al., 2004; Masoud and Jespersen, 2006; Silva et al., 2008; Silva, 2014; Evangelista et al., 2015; Pereira et al., 2015; Elhalis et al., 2020b). These features make yeasts promising candidates as starter cultures in the primary processing of coffee beans. Recently, a few trials of inoculated coffee wet fermentation using yeasts such as *S*. *cerevisiae*, *Torulaspora*, and *P. fermentans* as starter cultures have been reported, mainly in Brazil, with promising results (Pereira et al., 2014, 2015; Martins et al., 2019). These studies reported increases in essential volatiles such as ethyl acetate, isoamyl acetate, ethanol, and acetaldehyde and improvement in sensory quality of the final products. Previously, we reported the crucial role of yeasts in the wet fermentation of Australian coffee beans and their contribution to the flavor, aroma, and overall sensory perceptions of high quality coffee (Elhalis et al., 2020b, 2021b). Subsequently, we studied the contribution of the isolated yeasts and found among the isolates, *H*. *uvarum* (MF574306.1) and *P. kudriavzevii* (CP021092.1) have shown high resistance to the stress conditions prevailing in coffee fermentation, and production of desirable flavor and aroma profiles (Elhalis et al., 2021a). In this study, these two yeasts were used, for the first time, as starter cultures to conduct inoculated coffee wet fermentation. The main objective of the study was to investigate the performance of the two yeasts in coffee wet fermentation in terms of their growth, metabolic activities and contribution to the sensory quality of the final coffee product, with a view to evaluating their potential as starter cultures. The second objective of this study was to verify the crucial positive roles of yeasts in coffee fermentation and coffee beverage quality as reported in our previous study where the growth of yeasts was suppressed by the addition of Natamycin and the resultant beans were compared to non-treated spontaneous fermentation (Elhalis et al., 2020b). The levels of key metabolites and volatiles were lower in the yeast suppressed fermented beans in addition to their lower sensory scores than that with yeast growth. Therefore we conclude that yeasts play crucial roles in fermentation for producing high quality coffee beans (Elhalis et al., 2020b).

## Materials and Methods

### Preparation of Yeast Starter Cultures

The yeasts *H. uvarum* and *P. kudriavzevii* were previously isolated from wet fermentations of coffee beans conducted at the laboratory of University of New South Wales, Sydney, NSW, Australia, and their classification was confirmed by sequencing of the 5.8S-ITS rDNA gene region (Elhalis et al., 2020a). Pure cultures of the yeasts were kept on malt extract agar (MEA) slants at 4°C from which they were inoculated into 250 ml of yeast extract broth (YE), incubated in shaking incubator (120 rpm) at 30°C overnight. The cultures were transferred into YE broth (750 ml) and incubated with shaking for 24 h at 30°C. Yeast cells were collected by centrifugation (3,200×*g*, 15 min) and resuspended in 200 ml 0.1% sterile peptone water (Sigma, Sydney, NSW, Australia). Cell counts in the suspensions were performed using a counting chamber (Improved Neubauer, Assistent, Germany) and further confirmed by plate counting on MEA.

### Inoculated Coffee Bean Fermentation With Yeasts

Freshly harvested coffee cherries (Coffea arabica var. Bourbon, 40 kg) from Zentveld’s Coffee Farm located in Newrybar, NSW, Australia were packed in polystyrene foam containers with ice and immediately airfreighted to UNSW Sydney. Details of sample preparation and fermentation were given in Elhalis et al. (2020a), and only experiments involved the inoculation of the yeast starter cultures are described here. De-pulped coffee beans (5 kg) were transferred to a sterile plastic box (56 × 42 × 33 cm) containing 15 L of tape water, which was inoculated with the suspension of *H. uvarum* and *P. kudriavzevii* separately and together with a 1:1 ratio. The initial population of each species in the ferment was adjusted to a total concentration of about 10^8^ cells/ml, which was allowed to ferment for 36 h at room temperature. The fermenting beans were mixed every 12 h. Samples (200 ml) of the liquid fraction of the ferments were collected every 12 h, which were either examined immediately by traditional culture dependent methods or frozen at −20°C until analysis by culture independent molecular methods. Samples of coffee beans (100 g) were also taken and frozen at −20°C for chemical analyses. Several preliminary fermentations were conducted to optimize the fermentation conditions and the initial inoculum counts. Two independent fermentations were conducted for each yeast species and the two yeasts combined together with a total of six inoculated fermentations were conducted. Another 2 × 5 kg of the de-pulped beans were fermented in exactly the same way except without the yeast inoculation (spontaneous fermentation), which was used as the control. After fermentation, coffee beans were dried and roasted as described in Elhalis et al. (2020b). The dried coffee beans were stored at −20°C in vacuumed plastic bags until further analysis. A laboratory dehuller was used to dehull the dried beans and 100 g samples were roasted in a laboratory roaster (IKAWA, London, United Kingdom) following to the procedures of the Specialty Coffee Association of America (SCAA, 2019). Roasting was performed at 225°C for 7 min and the air flow was set at high, which flowed from the bottom to the coffee beans separating the lightweight undesirable beans. The roasted beans were collected and ground using an electric coffee grinder (Mahlkonig’s EK43, Revesby, Sydney, NSW, Australia), which was cleaned between samples. Ground coffee samples were vacuum packed in aluminum bags and stored at −20°C until further analysis.

### Sensory Analysis

Sensory evaluation (Cup test) of the coffee was conducted as described previously in Elhalis et al. (2021b) by a panel of three expert coffee testers with Q-Grader coffee certification (Coffee Logic Institute, Sydney, NSW, Australia). In brief, the testing protocol consisted of a three-step process: first, the aroma evaluation of the dry grounded coffee beans by sniffing; second, the aroma evaluation of brewed coffee by sniffing over the cup with stirring three min after its preparation; and third, evaluation of the aroma after 8–10 min of brewing. Each coffee sample was assessed in five cups. Apart from the aroma, coffee characteristics such as acidity, balance, body, aftertaste, uniformity, sweetness, clean cup, and overall impression were also evaluated by tasting the brew. The total score of each sample was estimated by the sum of all the attributes. At the end, the members of the panel were also asked to describe the specific flavor of each sample.

### Microbiological Analysis

#### Culture Dependent Methods

The populations of the inoculums and the endogenous yeasts, as well as total aerobic bacteria count (TABC) and lactic acid bacteria (TLAB) were monitored by traditional plate count methods using appropriate agar media. Details of the methods were given in Elhalis et al. (2020a). Wet mounts were used to differentiate the cellular morphologies of the isolates. LABs were further confirmed by Gram staining and catalase test. The reported population data are the means of duplicate analyses within a standard deviation of ±0.05. For the inoculated yeasts, their identities were initially based on the colony morphology, growth characteristics and the 5.8S ITS rRNA gene region sequencing as described previously in Elhalis et al. (2020a). During the fermentation processes the identity of the inoculated yeasts were further confirmed by restriction fragment length polymorphism PCR (PCR-RFLP) of the 5.8S-ITS rDNA using primers ITS1 (5′-TCCGTAGGTGAACCTGCGG-3′) and ITS4 (5′-TCCTCCGCTTATTGATATGC-3′) that were further digested using *Cfo*I, *Hae*III, *Cfo*I, and *Hin*fI (White et al., 1990; Esteve-Zarzoso et al., 1999). Specifically, YEA plates with 30–300 colonies were selected and pure colonies were picked randomly to extract DNA (Cocolin et al., 2002). PCR reactions were started by initial denaturation at 98°C for 5 min and 35 cycles of denaturation at 98°C for 30 s, followed by annealing at 56°C for 30 s, extension at 72°C for 50 s, and final extension at 72°C for 7 min. The PCR products were digested using *Cfo*I, *Hae*III, *Cfo*I, and *Hin*fI. The molecular sizes of the PCR and restriction fragment products were estimated by gel electrophoresis on 1.5 and 3% agarose gels, respectively, and compared with yeast profiles reported in the literature to confirm the identity of the yeasts (Esteve-Zarzoso et al., 1999; del Monaco et al., 2014).

#### Quantitative Real-Time PCR Analysis of Yeasts

##### Direct DNA Extraction From the Fermentation Mass

Samples (10 ml) were aseptically taken from three different points (surface, middle and bottom) of the fermentation mass and first centrifuged at low speed (200×*g*, 3 min) to precipitate the coarse particles and the supernatants were collected. The precipitates were further washed twice with sterile 50 mM phosphate buffer (pH 6.0) to recover the yeast cells that might precipitate with the coarse particles during the centrifugation. All the supernatants from the first centrifugation and the washing steps were transferred to fresh tubes and centrifuged in an EBA12 Centrifuge (Hettich, Newport Pagnell, Buckinghamshire, United Kingdom) at 13,000 rpm for 10 min to precipitate the microbial cells and the supernatant discarded. DNA from the cells were extracted using a three-step procedure following Elhalis et al. (2020a). In brief, the cell pellet was resuspended in 200 μl of 50 mM phosphate buffer (pH 6.0) containing cell lysis enzymes (chitinase 500 mU/ml and lyticase 0.2 U) and incubated at 30°C for 3 h. The enzymatically digested cells were further lysed using 200 μl of breaking buffer (2% Triton X-100, 1% SDS, 100 mM NaCl, 10 mM *Tris*–Cl pH 7.6, and 1 mM EDTA pH8) and vortex for 1 min. Finally, the mixtures were sonicated for 1 h at room temperature. The extracted DNA was precipitated by adding an equal volume of isopropanol and purified with the DNeasy blood and tissue kit (Qiagen, Venlo, Netherlands) according to the manufacturer’s instructions. The concentrations of extracted DNA and protein contamination were quantified by a spectrophotometer at UV 260 and 280 nm, respectively.

##### Design of Primers and Determination of Annealing Temperature

The specific primers were created in our laboratory using Primer3 (Rozen and Skaletsky, 2000) and their specificity were check using the BLAST algorithm (National Center for Biotechnology Information, ML, United States^1^). One pair of the specific primers was created for each target yeast species (Table 1). The annealing temperature of each primer pair was determined by performing temperature gradient PCR of 50–60°C. PCR was performed in a thermocycler (C1000 Thermal Cycler, Bio-Rad, Hercules, CA, United States) in 25 μl final volume using Hot Start Taq 2X Master Mix (New England Biolabs, Ipswich, MA, United States) according to manufacturer’s instructions. The PCR conditions consisted of an initial step at 98°C for 5 min, followed by 35 cycles of denaturation at 98°C for 30 s, annealing at temperature gradient of 50–60°C for 30 s, and extension at 72°C for 50 s. A final extension was conducted at 72°C for 7 min. The PCR products were analyzed by electrophoresis on 3% agarose gel in 1X TBE buffer. The optimal annealing temperatures obtained are given in Table 1. The specificity of the primers was further verified using target yeasts and non-target fungal DNA (*S*. *cerevisiae*) as described above.

**TABLE 1:** Correlation coefficient, slope, and efficiency of standard curves estimated from serial dilution of the target yeasts grown in YE broth.

Species	Primer	Sequence 5′–3′	*R* ^2^	Slope	Efficiency* (%)
*Hansinaspora uvarum*	H.U.J.F	TTGGTTGTGGGCGATACTCA	0.977	−3.986 ± 0.018	78.187 ± 3.77
	H.U.H.R	CATCACAGCGAGAACAGCGT			
*Pichia kudriavzevii*	P.K.J.F	TTGAGCCGTCGTTTCCATCTT	0.998	−3.663 ± 0.118	87.508 ± 0.641
	P.K.H.R	CAGCGGGTATTCCTACCTGA			

*Efficiency was estimated by the formula E = 10^–1/slope^ – 1 (Higuchi et al., 1993).

##### Construction of Standard Curves

The yeast species were cultivated in YE broth at 30°C for 24 h. Final cell counts were determined by plate counting on YEA in duplicate. DNA was extracted as described above from the 24 h culture broth serially diluted with TE buffer (pH 8.0) from 10^12^ to 10^3^ cells/ml. PCR assays were carried out using iTaq Universal SYBR Green Supermix (New England Biolabs, Ipswich, MA, United States) according to the manufacturer’s instructions. A linear relationship was created by plotting the cell counts against the cycle threshold values (Carrasco et al., 2019). The slope of the curve was used to estimate the amplification efficiency following the equation *E* = 10^–1/slope^ − 1 (Higuchi et al., 1993) as shown in Table 1. The specificity of the primers in qPCR was also confirmed by conventional PCR using target and non-target yeasts as described above. The experiment was repeated twice each with three replicates.

##### Quantitative PCR of Target Yeast Populations During the Wet Fermentations

The extracted DNA from the fermentation mass were amplified using iTaq Universal SYBR Green Supermix (New England Biolabs, Ipswich, MA, United States) according to the manufacturer’ instructions in the same way as described above. The *C*_*t*_ was calculated automatically by the instrument and the cell concentration of each yeast was estimated by using the calibration curve for each target yeast constructed as described above. The experiment was repeated independently two times and each assay was done in triplicates and non-template controls were included in all PCR runs.

### Chemical Analysis

Details of the methods used for analysis of sugars, organic acids, glycerol, and mannitol in the green beans were described in Elhalis et al. (2020a). Volatile compounds in the green and roasted beans were analyzed by head space solid phase microextraction gas chromatography mass spectrum (HS-SPME/GCMS) according to Frank et al. (2017) and details of the method was described in Elhalis et al. (2020b). Volatile compounds were identified by matching their electron impact mass spectra with those of reference compounds in the NIST mass spectral library and by comparing linear retention indices with published values in the NIST and PubChem websites. Further confirmation was done in some cases using pure reference standards. The concentration of the volatiles was determined semi-quantitatively using the Shimadzu proprietary software “LabSolutions” (Version 2.53). The extraction and analysis of each sample were performed in triplicates and the results were expressed as an average (±standard deviation).

### Statistical Analysis

One-way ANOVA was carried out to compare means between sample treatments, and Tukey’s HSD *post hoc* test was used to separate means of significant differences. Differences were statistically significant at *p* < 0.05. All statistical analyses were performed with GenStat^®^ (16th Edition, VSN International, Hemel Hempstead, United Kingdom).

## Results

### Microbial Ecology

**FIGURE 1:**
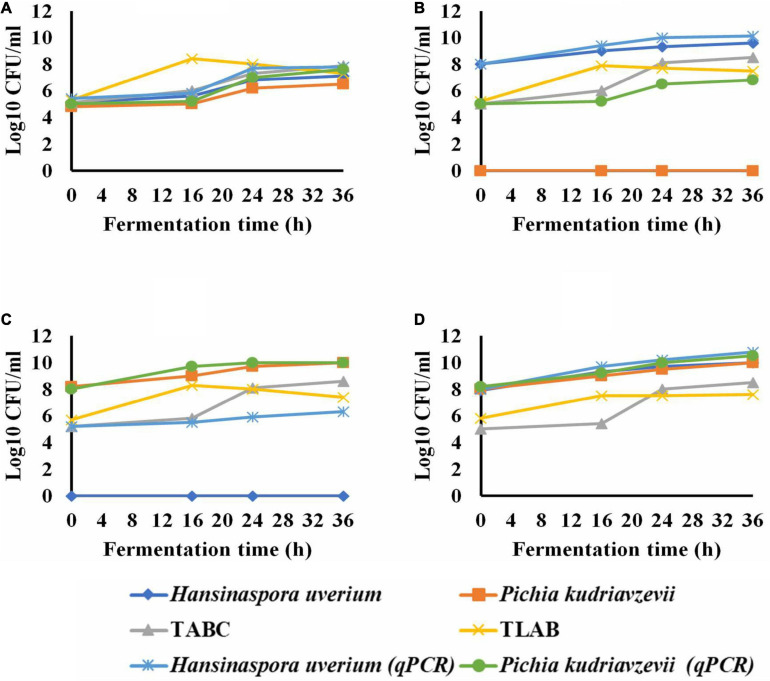
The growth of yeasts during wet coffee beans fermentation; spontaneous (control) **(A)**; *Hansinaspora uvarum* inoculations **(B)**; *Pichia kudriavzevii* inoculations **(C)** and inoculation with *H*. *uvarum* and *P. kudriavzevii* Combined **(D)**. TABC, total acrobic bacteria count; TLAB, total lactic acid bacteria; qPCR, quantitative real time PCR. Microbial populations were determined by plate coubting with SD of means less than 5% (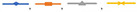) and qPCR with SD of means ranged from 0.006 to 0.09 log10 CFU/ml (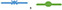).

The microbial population showed a total yeast count of 5.5 log CFU/ml during the first hour of the spontaneous fermentation that grew to 7.3 log CFU/ml within 36 h. *H. uvarum*, *P. kudriavzevii* were the most abundant endogenous yeast isolates with an initial population of 5.1 and 4.8 log CFU/ml, respectively (Figure 1A). As the fermentation progressed, *H. uvarum and P. kudriavzevii* grew to maximum populations of 7.1 and 6.5 log CFU/ml, respectively. The initial total aerobic bacterial and lactic acid bacteria counts were 5.1 and 5.3 log CFU/ml which subsequently grew to 7.9 and 7.3 log CFU/ml, respectively, in the spontaneous fermentation. The qPCR analysis results for *H. uvarum* and *P. kudriavzevii* of the spontaneous fermentation were similar to the plat counts.

In the fermentation inoculated with *H. uvarum*, all the colonies appeared on YEA agar plates were *H. uvarum* with an initial count of 8.0 log CFU/ml which subsequently grew to a maximum population of 9.6 log CFU/ml within 36 h (Figure 1B). The growth pattern of total LAB in the fermentation was similar to that in the spontaneous fermentation. The total aerobic bacterial count at the beginning was 5 log CFU/ml, which grew to a maximum population of 8.5 log CFU/ml within 36 h. The qPCR results showed an initial count of *H. uvarum* of 8 log CFU/ml, which subsequently increased to a maximum population of log 10.1 CFU/ml as the fermentation progressed. The qPCR also detected *P. kudriavzevii* throughout the entire *H. uvarum* inoculated fermentation with an initial count of 5.0 log CFU/ml that grew to a maximum population of 6.3 log CFU/ml at the end.

In the fermentation inoculated with *P. kudriavzevii*, 100% of the colonies grown on YEA agar plates were *P. kudriavzevii*, with the populations ranging from 8.2 to 10 log log CFU/ml, and no other yeast species were detected. The total aerobic bacterial and LAB populations and their growth dynamics were similar to those in the *H. uvarum* inoculated fermentation. The qPCR identified *P. kudriavzevii* as well as *H. uvarum* in the fermentation with initial populations of 5.2 and 8.0 log CFU/ml, which subsequently grew to maximum populations of 6.3 and 10.0 log CFU/ml, respectively (Figure 1C).

In the fermentation inoculated with both yeasts, 100% of the recovered yeasts on YEA were *H. uvarum* and *P. kudriavzevii* sharing about 50% each, which grew to maximum populations of 10.0 log CFU/ml each at the end. The total aerobic bacteria and LAB grew to maximum populations of 8.5 and 7.6 log CFU/ml, respectively. The qPCR results agreed with the plate counts at the start of the fermentations, and then *H. uvarum* and *P. kudriavzevii* grew to 10.8 and 10.5 log CFU/ml in at the end, respectively (Figure 1D).

### Microbial Metabolisms

#### Non-volatile Compounds

The concentrations of the major non-volatile compounds found in the mucilage and endosperm of coffee beans are shown in Tables 2, 3. Three main sugars were detected in coffee beans, which were sucrose, glucose and fructose. The levels of these sugars declined during the fermentation which were faster in the inoculated fermentation (*p* > 0.05). Lactic acid and glycerol were the major end metabolites detected during fermentations which were about 2-fold higher in the inoculated beans than the controls (*p* > 0.05).

**TABLE 2:** Comparison of chemical composition and microbial metabolites in the mucilage of coffee beans undergone spontaneous (control) and inoculated fermentations with *H. uvarum*, *P. kudriavzevii*, and the two yeasts combined.

Compounds	Concentration (g/100 g)							
	Control		*H*. *uvarum*		*P*. *kudriavzevii*		Combined yeasts	
	*T0*	*T36*	*T0*	*T36*	*T0*	*T36*	*T0*	*T36*
Sucrose	13.03 ± 1.50^a^	2.01 ± 0.17^b^	13.70 ± 0.75^a^	1.02 ± 0.20^c^	13.70 ± 3.10^a^	1.22 ± 0.02^c^	13.18 ± 0.17^a^	ND^d^
Glucose	19.41 ± 1.20^a^	7.06 ± 2.07^b^	21.09 ± 1.01^a^	ND^d^	21.80 ± 0.79^a^	2.01 ± 0.08^c^	27.20 ± 1.04^a^	2.03 ± 0.03^c^
Fructose	27.02 ± 2.01^a^	4.05 ± 0.08^b^	27.04 ± 1.33^a^	3.12 ± 0.07^c^	27.70 ± 1.22^a^	3.03 ± 0.05^c^	27.14 ± 1.14^a^	2.70 ± 0.04^c^
Glycerol	ND^d^	0.90 ± 0.02^c^	ND^d^	1.70 ± 0.03^b^	ND^d^	1.32 ± 0.02^b^	ND^d^	2.10 ± 0.13^a^
Mannitol	ND^b^	0.92 ± 0.03^a^	ND^b^	1.10 ± 0.05^a^	ND^b^	1.00 ± 0.01^a^	ND^b^	1.22 ± 0.03^a^
Citric acid	1.18 ± 0.03^b^	0.80 ± 0.03^c^	1.23 ± 0.01^b^	1.50 ± 0.02^a^	1.16 ± 0.02^b^	0.83 ± 0.01^c^	1.20 ± 0.03^b^	1.63 ± 0.03^a^
Malic acid	2.30 ± 0.04^a^	1.56 ± 0.01^b^	2.12 ± 0.02^a^	1.53 ± 0.01^b^	2.11 ± 0.03^a^	1.51 ± 0.01^b^	2.13 ± 0.01^a^	1.52 ± 0.02^b^
Quinic acid	1.90 ± 0.03^a^	1.52 ± 0.02^b^	1.97 ± 0.01^a^	1.62 ± 0.02^b^	2.02 ± 0.08^a^	1.52 ± 0.02^b^	1.93 ± 0.04^a^	1.52 ± 0.01^b^
Succinic acid	2.14 ± 0.11^a^	1.32 ± 0.02^b^	2.05 ± 0.13^a^	1.43 ± 0.09^b^	2.09 ± 0.08^a^	1.36 ± 0.05^b^	2.14 ± 0.10^a^	1.53 ± 0.01^b^
Lactic acid	ND^c^	0.81 ± 0.02^b^	ND^c^	1.63 ± 0.07^a^	ND^c^	1.52 ± 0.02^a^	ND^c^	1.73 ± 0.06^a^

*Means in each row bearing the same letters are not significantly different (p > 0.05) from one another using Tukey’s test (mean ± standard deviation).*

**TABLE 3:** Comparison of chemical composition and microbial metabolites in the endosperm of coffee beans undergone spontaneous (control) and inoculated fermentations with *H. uvarum*, *P. kudriavzevii* and the two yeasts combined.

Compound	Concentration (g/100g)							
	Control		*H*. *uvarum*		*P*. *kudriavzevii*		Combined yeasts	
	*T0*	*T36*	*T0*	*T36*	*T0*	*T36*	*T0*	*T36*
Sucrose	10.80 ± 0.50^a^	5.00 ± 0.71^b^	10.90 ± 0.35^a^	5.06 ± 0.28^b^	12.13 ± 0.33^a^	4.93 ± 0.49^b^	10.71 ± 0.21^a^	4.50 ± 0.47^b^
Glucose	0.92 ± 0.01^a^	0.84 ± 0.01^b^	0.93 ± 0.03^a^	0.67 ± 0.01^c^	0.88 ± 0.02^a^	0.62 ± 0.01^c^	0.93 ± 0.01^a^	0.63 ± 0.02^c^
Fructose	1.82 ± 0.24^a^	0.93 ± 0.11^b^	1.85 ± 0.17^a^	0.71 ± 0.09^b^	1.52 ± 0.21^a^	0.84 ± 0.10^b^	1.34 ± 0.22^a^	0.73 ± 0.07^b^
Glycerol	ND^c^	0.09 ± 0.01^b^	ND^c^	0.20 ± 0.03^a^	ND^c^	0.17 ± 0.09^a^	ND^c^	0.21 ± 0.03^a^
Mannitol	ND^b^	0.10 ± 0.01^a^	ND^b^	0.09 ± 0.01^a^	ND^b^	0.08 ± 0.02^a^	ND^b^	0.09 ± 0.01^a^
Citric acid	1.40 ± 0.01^a^	1.21 ± 0.02^b^	1.31 ± 0.01^a^	1.12 ± 0.02^b^	1.29 ± 0.06^a^	1.00 ± 0.02^b^	1.36 ± 0.01^a^	1.20 ± 0.03^b^
Malic acid	0.42 ± 0.03^a^	0.23 ± 0.10^b^	0.44 ± 0.02^a^	0.15 ± 0.04^b^	0.40 ± 0.07^a^	0.21 ± 0.01^b^	0.41 ± 0.05^a^	0.20 ± 0.03^b^
Quinic acid	0.67 ± 0.02^a^	0.53 ± 0.01^b^	0.71 ± 0.01^a^	0.51 ± 0.03^b^	0.70 ± 0.02^a^	0.52 ± 0.04^b^	0.78 ± 0.03^a^	0.49 ± 0.01^b^
Succinic acid	0.37 ± 0.02^a^	0.23 ± 0.01^b^	0.39 ± 0.04^a^	0.22 ± 0.02^b^	0.33 ± 0.07^a^	0.21 ± 0.03^b^	0.34 ± 0.03^a^	0.20 ± 0.01^b^
Lactic acid	ND^c^	0.41 ± 0.13^b^	ND^c^	0.83 ± 0.17^a^	ND^c^	0.78 ± 0.12^a^	ND^c^	0.81 ± 0.09^a^

*Means in each row bearing the same letters are not significantly different (p > 0.05) from one another using Tukey’s test (mean ± standard deviation).*

The concentrations of the major non-volatile compounds found in the mucilage layers of coffee beans are shown in Table 2. The mucilage layers contained three sugars, which were fructose (27.0 g/100 g), glucose (19.4 g/100 g) and sucrose (13.0 g/100 g). The levels of these sugars declined during the spontaneous (non-inoculated control) fermentation to 2–7 g/100 g. In the inoculated fermentations, the decreases of sugars were faster and more extensive, leading to either total consumption or lower concentrations than those in the control. Four main organic acids were detected at the beginning of the fermentations, which were malic, succinic, quinic, and citric with concentrations of about 1–2 g/100 g. During the control fermentation, their concentrations declined to about 0.8–1.6 g/100 g. Similar trends were observed in the inoculated fermentations; however, the level of citric acid increased slightly to a maximum concentration of about 1.6 g/100 g in fermentations inoculated with *H. uvarum* and combined yeasts. Furthermore, lactic acid was not present in any of the fermentations at the start, and was first detected at 16 h with a concentration of about 0.4 g/100 g. The level of lactic acid increased in the control beans to 0.8 g/100 g, while in the inoculated fermentations it was about twofold higher than in the former. Similarly, the mucilage did not contain glycerol at the beginning of the control fermentation and was first detected at 24 h with a value of 0.7 g/100 g which subsequently increased to a maximum concentration of 0.9 g/100 g at the end. In the inoculated fermentations, glycerol was detected earlier at 16 h, which increased to maximum concentrations of 1.3–2.1 g/100 g. For all fermentations, mannitol was not present at the beginning, and was first detected at 16 h with a concentration of about 0.4 g/100 g, then increased to maximum concentrations of 0.9–1.2 g/100 g, without significant differences between the different fermentations.

Less extensive changes in these non-volatile compounds were observed in the endosperm compared to the mucilage layer, Table 3. Sucrose, fructose, and glucose were detected in the endosperm at the start of fermentation with the concentrations of 10.8, 1.8, and 0.9 g/100 g, respectively. In the control fermentation, sucrose gradually declined to 5.0 g/100 g, while fructose and glucose reached approximately 0.9 g/100 g each. The sugar metabolism was faster in the inoculated fermentations where they ended up with slightly lower concentrations compared to the spontaneous fermentation. Four main organic acids were detected in the endosperm at the beginning of the fermentations with citric acid being the major one, followed by quinic, malic and to by 0.4–1.4 g/10 g. No significant differences in the concentration of these organic acids were observed in the inoculated fermentations. Although the level of citric acid detected in the mucilage layer increased during the inoculated fermentations, no corresponding change was observed in the endosperm. Lactic acid was first detected at 16 h, which subsequently increased to a maximum concentration of 0.8 g/100 g in the inoculated fermentations, which doubled the level of the control. Glycerol and mannitol were not present at the beginning of all fermentations and was first detected at 24 h, which gradually increased to a maximum concentration of about 0.1 g/100 g each in the control. No significant difference was observed in the concentration of mannitol in the inoculated fermentations; however, glycerol was detected earlier, and its concentration was approximately twice that of the control.

#### Volatile Compounds

Seventy eight (78) volatile compounds were identified in the coffee beans, which were grouped based on their chemical class into alcohols, aldehydes, ketones, esters, acids, phenols, furans, pyrazines, sulfur containing compounds and miscellaneous (Supplementary Table 1).

##### Alcohols

Nine main alcohols were identified in the green beans with a total concentration of 1,166.5 μg/kg, which represented about 43% of the total volatiles in the beans. Ethanol was the most abundant component with a maximum concentration of 481.7 μg/kg, followed by isopentyl alcohol, 3-methyl-2-buten-1-ol, isoamyl alcohol, 1-non-anol, 3-hexanol, and phenyl ethyl alcohol. In the inoculated fermentations with *H. uvarum* and the two yeasts combined, ethanol concentration was approximately threefold higher than that of the control, while in the *P. kudriavzevii* inoculated fermentation it was fourfold higher than the control. The level of isoamyl alcohol was more than threefold higher in the fermentation inoculated with *H. uvarum* and about fourfold higher with *P. kudriavzevii* and the combined yeasts, compared to that in the spontaneous fermentation (*p* < 0.05). Similarly, the phenylethyl alcohol level was approximately twofold higher in the fermentation inoculated with *H. uvarum*, fivefold with *P. kudriavzevii* and sixfold with the combined yeasts, compared to the control (*p* < 0.05). No significant differences were detected with the remaining alcohols among the different green bean types (*p* > 0.05). As expected, after roasting the concentrations of total alcohol in the various types of beans decreased to about half of those in the green beans; however, the levels of residue ethanol, isoamyl alcohol, and phenylethyl alcohol were significantly higher (2–10 folds) in the inoculated fermentations than those in the control fermentation (*p* < 0.05). Furthermore, four additional alcohols were identified in the roasted beans but not in the green beans, which were 2-methyl-3-buten-2-ol, 2,3 butanediol, 2,3-hexanediol, and 1,4-butanediol, with no significant differences in their concentrations among the different types of beans (*p* > 0.05). Low amounts of furfuryl alcohol and 1-non-anol were detected in green beans, which increased significantly after roasting with maximum concentrations of 115.5 and 289.3 μg/kg in the control beans (*p* < 0.05), respectively. The level of furfuryl alcohol was significantly higher in the inoculated fermentations, especially with combined yeasts, while the 1-non-anol level was similar in all bean types (*p* > 0.05).

##### Aldehydes

Aldehydes represented about 10% of the total volatiles in the green beans with a maximum total concentration of 256.3 μg/kg, which subsequently increased by about sevenfold after roasting in the control beans. Acetaldehyde, hexanal, and benzaldehyde were the most abundant compounds among this group and their levels were generally higher in the green beans from inoculated fermentations compared to the control (*p* < 0.05). After roasting, the concentration of acetaldehyde remained largely unchanged in all fermentations; however, its level was significantly higher in the beans from inoculated fermentations, especially with *P. kudriavzevii*, than that in the control beans (*p* < 0.05). Similar patterns were also observed for hexanal, while benzaldehyde was not detected in any of the roasted beans Furthermore, neo-formation of aldehydes were detected in the roasted beans and these were furfural and 5-methyl furfural, with no significant differences observed among the different bean types (*p* > 0.05). Low levels of 3-methyl butanal was identified in all green beans with similar concentrations which subsequently increased after roasting with significantly higher concentrations in the inoculated beans, especially with combined yeasts, than those in the control beans (*p* < 0.05).

##### Esters

About 30% of the total volatiles quantified in the green beans were esters, among which ethyl acetate (52%) and methyl acetate (46%) were the most abundant in the control fermentation. Higher levels of ethyl acetate and methyl acetate were observed in the inoculated fermentations, especially with *H. uvarum* and combined yeasts, compared to the control fermentation (*p* < 0.05). Furthermore, trace amounts of methyl formate was also detected in the green beans, and its levels were significantly higher in the inoculated fermentations than that in the control (*p* < 0.05). After roasting, the levels of the esters declined; however, their concentrations remained significantly higher in the inoculated fermentations than in the control (*p* < 0.05). Furthermore, three additional esters were detected after roasting but not identified in the green beans, namely furfural propionate, furfuryl formate, and furfuryl acetate. Significantly higher levels of furfuryl formate and furfuryl acetate were observed in the inoculated fermentations than that found in the control (*p* < 0.05), while the concentrations of furfuryl propionate were almost the same in all bean types (*p* > 0.05).

##### Ketones

The fermented green beans contained low levels of ketone compounds, which increased by about 14-fold after roasting in the control fermentation. Three main ketones were detected in the green beans, namely 2-butanone, 3-pentanone, and 2,3-butanedione. No significant differences in the concentration of 2-butanone were found between the fermentations (*p* > 0.05), while the levels of 3-pentanone and 2,3-butanedione were significantly higher in the inoculated fermentations compared to the control (*p* < 0.05). After roasting, the concentration of 3-pentanone declined, while those of 2,3-butanedione and 2-butanone increased, and their levels remained higher in the inoculated fermentations than that of the controls (*p* < 0.05). Furthermore, three additional ketones were formed after roasting, namely 2,3-pentanedione, 2,3-hexanedione, and 2,3-heptanedione. No significant differences were found in the concentration of 2,3-heptanedione between the fermentations, while the levels of 2,3-pentanedione and 2,3-hexanedione were significantly higher in the inoculated fermentations, especially with the combined yeasts, compared to the control fermentation (*p* < 0.05).

##### Acids and Phenols

Two main organic acids were detected in the green beans, which were acetic (383.1 μg/kg) and butanoic (20.1 μg/kg) acids in the spontaneous control fermentation. In the inoculated fermentations, the level of acetic acid was approximately double while butanoic acid was half of that found in the control beans (*p* < 0.05). After roasting, the concentrations of both acetic and butyric acids declined, however, the level of the former was slightly higher in the inoculated fermentations, especially with combined yeasts, while the level of butanoic acid was lower than that in the control (*p* < 0.05). Low levels of phenol were detected in the green beans of all fermentations with similar concentrations, which remained relatively unchanged after roasting. In addition, three new phenols were formed during roasting, namely 2-methoxy phenol (guaiacol), 2-methyl phenol, and 2-methoxy-4-vinylphenol but their concentrations were not significantly different among the different types of beans (*p* > 0.05).

##### Pyrazines, Pyrroles, and Pyridines

Pyrazine compounds were detected in only the roasted beans and not in the green beans from all fermentations. A total of 12 pyrazines, representing about 27% of the total volatiles, were detected after roasting, among which 2-methylpyrazine, 2,5-dimethypyrazine, 2,6-dimethylpyrazine, 2,3-dimethylpyrazine, and pyrazine, 2-ethyl-6-methyl were the most abundant. The concentrations of these volatiles, however, were significantly higher in the inoculated fermentations, especially with *P. kudriavzevii* and the combined yeasts, compared to those in the control. Roasted beans also contained significant amounts of 2-ethyl-5-methylpyrazine; however, no significant differences were detected between the different fermentations (*p* > 0.05). Four main pyrroles were identified in the roasted beans, namely 1-methyl-pyrrole, 2-ethyl-pyrrole, 2-formyl-1-methyl pyrrole, and 2-formyl pyrrole. The concentration of 2-formyl pyrrole was significantly higher in the control and *H. uvarum* inoculated fermentation compared with other fermentations. The levels of 2-ethyl-pyrrole and 1-methyl-pyrrole were significantly higher in the inoculated fermentations compared to the control (*p* < 0.05). No significant differences in the concentration of 2-formyl-1-methylpyrrole were observed among the different fermentations (*p* > 0.05). Three pyridine compounds were also identified in the roasted beans, among which pyridine was the most abundant, and its level was significantly higher in the inoculated fermentations, especially with *P. kudriavzevii* and combined yeasts, than in the control (*p* < 0.05).

##### Furans and Sulfides

A total of 10 furan compounds were detected after roasting, but not identified in the green beans, representing about 4% of the total volatiles, among which 2-acetylfuran and 2-propionylfuran were the most abundant. The concentration of 2-acetylfuran was higher in the inoculated fermentations than that of the control (*p* < 0.05), while no significant difference was detected in the level of 2-propionylfuran in all sample types. In addition, significant higher levels of 2-methylfuran, 2,5-dimethylfuran, 2-allylfuran, and tetrahydro-2-(methoxymethyl) furan were found in the inoculated beans. Sulfur containing volatiles were not detected in any green beans but identified after roasting. A total of six compounds were detected, among which dimethyl trisulfide was the most abundant followed by furfuryl methyl sulfide, dimethyl disulfide, methanethiol, dimethyl sulfide, and bis-2-(furfuryl)-disulfide with similar concentrations in all fermentations (*p* > 0.05).

### Sensory Analysis

**FIGURE 2:**
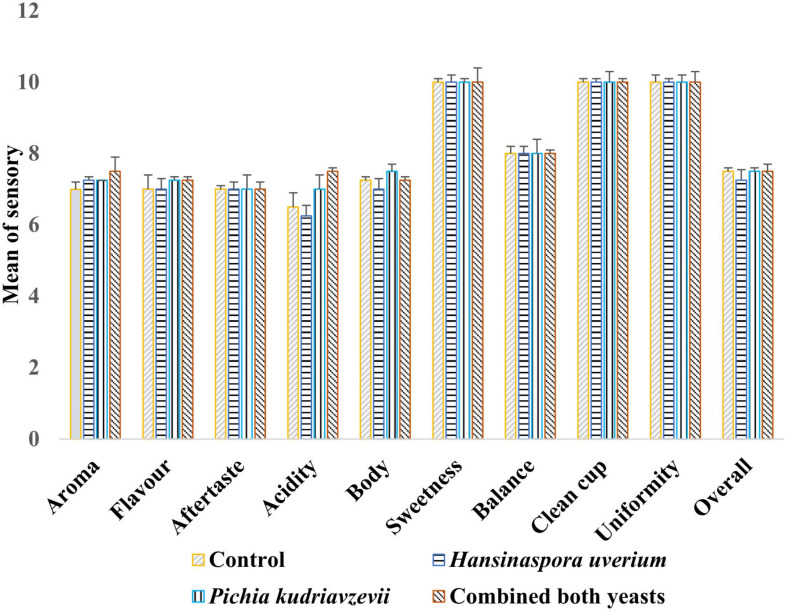
Scores of cup test means (SCAA) by three Q-Graded coffee panelists for coffee beverages produced with roasted beans from the three inoculated (*H. uvarum, P. kudriavzevii*, combined yeasts) and spontaneous (control) fermentations (Mean ± standard deviation).

Results of cup tests by three Q-Grade coffee masters showed no statistically significant differences between coffees brewed from beans from different fermentations on sweetness, balance, clean cup, uniformity, aftertaste, and overall scores (Figure 2). Coffee produced from the inoculated fermentations, especially with *P. kudriavzevii*, was awarded high scores of flavor, aroma and acidity than the control fermentation. The maximum overall score (82.0) was awarded to coffee from the inoculated fermentation with combined yeasts (*p* < 0.05), while the score for *P. kudriavzevii* and *H. uvarum* inoculated fermentations and the control were 81.5, 79.8, and 80.3, respectively, although the differences in these scores were not statistically significant (*p* > 0.05). The panel reported distinctive sensory characteristics for each coffee beverage. The coffee beverage produced from the control fermentation was perceived to be “vanilla,” “plummy,” and fruity in aroma. The beverage produced from *H. uvarum* inoculated fermentation was characterized by “roasted almond” aroma. The coffee produced by *P. kudriavzevii* inoculated fermentation was described as “malty,” “vanilla,” peanut in flavor, while the coffee produced inoculated fermentation with combined yeasts were characterized by earthy, apple cider, and walnut notes and a smooth mouthfeel.

## Discussion

Primary coffee processing, especially the fermentation stage, is currently conducted overwhelmingly in the traditional way where fermentation occurs spontaneously. Previous studies have demonstrated the critical roles of yeasts in the spontaneous wet coffee fermentation process and their capability to improve the quality of the resultant beans by producing desirable metabolites and aromatic compounds (Elhalis et al., 2020a, b, 2021b). *H. uvarum* is known for its ability to quickly utilize available sugars present in the mucilage, which might help the mucilage degradation process (Pretorius, 2000). In wine fermentation, *H. uvarum* was reported to be present in the initial stage and produce high concentrations of acetic acid and its esters (Heard and Fleet, 1986; Peddie, 1990). *P. kudriavzevii* was reported to resist stresses such as acidic conditions, high temperature and high salt content, making it applicable for industrial fermentation processes (Koutinas et al., 2016; Techaparin et al., 2017; Chamnipa et al., 2018). In addition, *P. kudriavzevii* has been shown to potentially improve the quality of products such as cocoa, cheese and alcoholic beverages (del Monaco et al., 2014; Saerens and Swiegers, 2016; Pereira et al., 2017; Zheng et al., 2018). To date, *H. uvarum* and *P. kudriavzevii* have not been reported for inoculation in the wet fermentation of coffee beans.

One of the major differences between the yeast inoculated and the spontaneous fermentations was that the yeast population in the former was about 2 logs CFU/ml higher than in the latter. This occurred in the inoculated fermentations with *H. uvarum*, *P. kudriavzevii* and the combination of both yeasts. This showed that the inoculated yeasts not only can successfully grow but also dominate the fermentation, demonstrating their potential as starter cultures. Both *H. uvarum* and *P. kudriavzevii* were inoculated in this study. As both yeasts species were naturally part of the microflora present in the spontaneous wet fermentation process, their populations in the inoculated fermentations were the sum of both the inoculum and the endogenous yeast counts and, therefore, relatively high. Furthermore, the growth of both yeasts in the combined inoculation illustrated that they can successfully grow together and were not be suppressed by each other, demonstrating the suitability of both species to be used as a mixed species starter culture. In contrast, when *H. uvarum* and other yeasts such as *S. cerevisiae* and *P. kluyveri* were used together in cocoa bean fermentation, *S. cerevisiae* grew to became the dominant species while the growth of *H. uvarum* was largely suppressed (Batista et al., 2009). Previous studies on yeast inoculated fermentations did not obtain consistent results. Pereira et al. (2015) inoculated a single yeast species, *P*. *fermentans*, in wet fermentation with a concentration of 7.5 log CFU/ml, which increased during fermentation to reach a maximum population of 8.8 CFU/ml after 12 h. However, in another study where *S*. *cerevisiae* and *Torulaspora delbrueckii* were inoculated together in wet fermentation at an initial count of 4.2–5.3 log CFU/g, the yeast population gradually declined to less than 2 log CFU/g at the end of fermentation after 72 h (Martins et al., 2019).

Surprisingly, the population of TABC also increased substantially in the yeast inoculated fermentations to reach a maximum population of about 8.5 log CFU/ml after 24 h, which was higher than that in the spontaneous fermentation at 7.9 CFU/ml, while the population of LAB was similar in all the fermentations. This showed that the high yeast growth in the inoculated fermentations did not suppress the growth of TABC and LABs, and might even helped the growth of some bacteria, possibly by the production of essential nutrients such as amino acids, vitamins, and purines (Viljoen, 2006). The ability of the yeasts and bacteria to co-exist also points to the potential for using a combination of yeast and bacteria as starter cultures in coffee fermentation.

The microbial population during fermentation was determined by both the traditional plate counting and the qPCR methods, and it was found that the former generally underestimated the population by about one log. This discrepancy could be due to that the plate counting technique only measured viable microbial cells in the samples, while qPCR quantified the DNA of all cells including viable, damaged, stressed, and dead cells. Furthermore, the plating technique was unable to detect the endogenous yeasts present in the fermentations due to the high numbers of the inoculated yeasts, where after necessary serial dilutions, the former fall below the detection limit. Such limitations of the plate counting method demonstrated the value of using molecular methods such as qPCR in the study of complex microbial communities. In addition, such techniques will enable real time monitoring of the growth of target microorganisms during fermentation, which would be valuable in industrial situations. qPCR techniques have been used to monitor the growth of bacteria and fungi in spoiled and fermented foods (Hein et al., 2001; Blackstone et al., 2003; Bleve et al., 2003; Haarman and Knol, 2005). Because of their specificity, sensitivity as well as speed compared to plate counting, the potential of using such techniques in monitoring industrial fermentation processes have been highlighted (Nakayama et al., 2007; Hagi et al., 2010). However, it should be taking into consideration that overestimation could occur due to possible recovery DNA from dead cells.

The higher yeast populations in the inoculated fermentations were corresponded with a faster sugar consumption in the mucilage. Interestingly, the decrease in the sugar level inside the bean (endosperm) was also faster in the inoculated fermentations than in the control. One possible explanation could be the leaching out of the sugars from the endosperm to the surrounding environment during wet fermentation (Wootton, 1971), and the lower sugar concentrations in the mucilage of the beans in inoculated fermentations could lead to a faster leakage of sugars from the endosperm. Furthermore, yeast population was much higher in the inoculated fermentations, which could cause a faster removal of the mucilage layer from the surface of the beans, which in turn might improve the sugar leaching process. The level of the reducing sugars inside the beans has a potential impact on coffee aroma, flavor and color during subsequent roasting process where sugars were a key participant of Maillard reaction and caramelization (Fischer et al., 2001). The consumption of sugars in the mucilage was accompanied by the accumulation of microbial metabolites such as glycerol, mannitol and lactic acid. As expected, higher concentrations of glycerol and lactic acid were detected in the inoculated fermentations than in the control, which is liked due to the higher populations of yeasts in the inoculated fermentations as yeasts are not only responsible for producing glycerol, but also produces lactic acid (Komesu et al., 2017). Glycerol is commonly detected in yeast fermented products and has a sweet and smooth mouthfeel, which typically has a positive contribution to sensory quality (Swiegers et al., 2005). The presence of relatively high levels of glycerol in beans from inoculated fermentations was likely responsible for the higher scores in mouthfeel of the coffee prepared from them compared to that of the spontaneous fermentation. The level of mannitol was similar in the different fermentations, which is likely due to the similar LAB counts in all the fermentations as mannitol was reportedly produced mainly by LABs through fructose metabolism (De Vuyst et al., 2010; Saha and Racine, 2011). Mannitol is characterized by a favorable cool taste (De Vuyst et al., 2010; Saha and Racine, 2011), which may explain the similar scores of aftertaste awarded to coffees brewed from the different types of beans.

The much higher levels of acetic acid in the inoculated fermentations than in the control were also likely due to the higher yeast populations in the former as yeasts such as *H*. *uvarum* are known produce acetic acid by direct sugar metabolism (Swiegers et al., 2005). Presence of acetic acid in coffee has a pleasant clean and sweet taste at low concentrations but produces a vinegary and pungent taste when its concentration exceed 1 mg/ml (Bertrand et al., 2012). The presence of the high levels of organic acids, i.e., acetic and lactic acids, in the inoculated beans are likely the cause of the higher acidic scores for coffee brewed from them. In contrast, the level of butanoic acid was lower in the inoculated fermentations. Butanoic acid has an onion like flavor and is produced mainly by undesirable growth of contaminated microorganisms during fermentations (Amorim and Amorim, 1977). These findings indicate the potential role of the inoculated yeasts in suppressing the growth of these undesirable microorganisms and minimizing the formation of off flavor compounds.

Both the green and roasted beans from inoculated fermentations also had significantly higher levels of alcohols than the control, with ethanol, isoamyl alcohol, and phenylethyl alcohol being the main ones. These alcohols are produced by yeasts in a wide range of fermented foods and beverages (Tamang and Fleet, 2009; Ho et al., 2014; Batista et al., 2015; Pereira et al., 2015). Ethanol contributes to the beverage viscosity and solubility of volatiles, while isoamyl alcohol and phenylethyl alcohol are characterized by their desirable sweet and fruity flavors (Tamang and Fleet, 2009; Gamero et al., 2019). Similarly, the inoculated beans (both green and roasted), especially with *P. kudriavzevii*, also had significantly higher concentrations of aldehydes, such as acetaldehyde, than those in the control. Acetaldehyde is a well-known yeast metabolite, as an intermediate of ethanol production, and has an almond, fruity and sweet aroma (Rosca et al., 2016). Interestingly, the concentration of 3-methyl butanal increased almost 100 times after roasting and was significantly higher in the inoculated beans, especially those with *P. kudriavzevii* and the combined yeasts. Branched-chain aldehydes such as 3-methyl butanal were detected in fermented food products and have malty and chocolate-like taste (Smit et al., 2009). Such aldehydes were mainly formed during heat treatment by Streaker degradation of amino acids with reducing sugars (Arnoldi et al., 1987). A substantial increase in the level of aldehydes, mainly acetaldehyde, was also observed with *P*. *fermentans* inoculated fermentations (Pereira et al., 2015). Regarding the esters, methyl acetate and ethyl acetate were the most important, whose concentrations were significantly higher in the inoculated green beans and remained more than two times higher after roasting in the inoculated beans, especially those with *H. uvarum* and combined yeasts than those in the control. Esters are considered key volatiles in many fermented foods and beverages even with low concentrations, and they are well known yeast metabolites (Peddie, 1990; Cristiani, 2001). Overall, our results showed that higher concentrations of alcohols and aldehydes were correlated with the growth of *P. kudriavzevii*, while higher levels of esters were observed with *H. uvarum.* Those metabolites, and also ketones, were detected with higher concentrations in the beans from inoculated fermentations with combined yeasts. Those volatiles are regarded as key volatiles that potentially contribute to coffee flavor and aroma (Czerny and Grosch, 2000), which may explain the high sensory scores of flavor and the distinct fruity and malty notes for coffee from the inoculated beans.

Most of the ketones, pyrazines, pyrroles, pyridines, furans, phenols, and sulfur containing volatiles were detected after roasting. These volatiles were usually generated by thermal reactions, such as Maillard reactions, of the bean components such as amino acids and sugars (Yaylayan and Keyhani, 1999; Daglia et al., 2007; Baggenstoss et al., 2008). These volatiles confers a wide range of sensory characteristics such as sweet, roast, caramel, buttery and earthy (Mayer et al., 2000). The significantly higher levels of ketones, pyrazines, and furans in the beans from inoculated fermentations suggest that yeast activities may have an impact on the internal components such as sugars and amino acids, which in turn affected the synthesis of these volatiles during roasting. However, as discussed above, beans from the inoculated fermentations had a lower concentration of reducing sugars. It was also reported that fermentation had no significant impact on the protein and peptide content of coffee bean (Ludwig et al., 2000; Elhalis et al., 2020a). Thus, direct yeast activities did not appear to explain their influence on the bean components. Pyrazines such as 2-ethyl-3,5-dimethylpyrazine were also found in coffee beans from *S*. *cerevisiae* and *T*. *delbrueckii* inoculated wet fermentation, but not detected in the beans from spontaneous fermentation (Martins et al., 2019). Yu and Zhang (2010) reported that the formation of volatiles during thermal treatments were dependent on the bean acidity. In addition, pretreating the beans with acetic acid to decrease the pH has been shown to facilitate the formation of pyrazines and furans during coffee roasting (Liu et al., 2019). Therefore, the higher concentrations of organic acids, such as lactic and acetic acids, in the inoculated green beans might be the reason for the higher concentrations of these volatiles in the beans from inoculated fermentations formed during roasting. However, further investigations are required to confirm this hypothesis.

## Conclusion

Results of this study showed that when *H*. *uvarum* and *P*. *kudriavzevii* were inoculated, either separately or together, into wet coffee bean fermentations, they were able to dominate the fermentations and became the overwhelmingly dominant microorganisms. Such process was shown to increase the concentrations of the final volatiles fractions of the roasted beans which were correlated with higher sensory scores for coffee brewed from them. The dominance of the inoculated yeasts led to faster and more complete utilization of sugars in the mucilage, with resultant production of higher concentrations of metabolites such as glycerol, alcohols, aldehydes, esters, organic acids, and pyrazines in the fermented green beans, compared with spontaneous fermentation. Although the levels of these metabolites were greatly reduced after roasting, they remained significantly higher in the beans from the inoculated fermentations, which were correlated with the distinct fruity notes reported with coffee brewed from them. Overall, the findings of this study confirmed the crucial role of yeasts in the wet fermentation of coffee beans, and their contribution to high quality coffee. In addition, this study also demonstrated that qPCR is a fast and reliable method for real time monitoring of yeast populations in fermentation. These results suggest that each yeast has distinctive metabolic activities that can be used individually or combined to modulate the coffee quality. Future studies could be directed to examine the potential of mixed cultures of yeast and bacterial species for accelerating coffee fermentation as well as improving product quality.

## Data Availability Statement

The original contributions presented in the study are included in the article/Supplementary Material, further inquiries can be directed to the corresponding author.

## Ethics Statement

The studies involving human participants were reviewed and approved by the HREAP Executives (HC number HC190689). Written informed consent for participation was not required for this study in accordance with the national legislation and the institutional requirements.

## Author Contributions

HE designed and conducted the experiments, performed the data analysis, and wrote the manuscript. JZ and DF supervised the experiments progress, interpreted the scientific values of the obtained data, and proofread the manuscript. JC contributed to supervision of the work. All authors read and approved the manuscript.

## Conflict of Interest

The authors declare that the research was conducted in the absence of any commercial or financial relationships that could be construed as a potential conflict of interest.

## Publisher’s Note

All claims expressed in this article are solely those of the authors and do not necessarily represent those of their affiliated organizations, or those of the publisher, the editors and the reviewers. Any product that may be evaluated in this article, or claim that may be made by its manufacturer, is not guaranteed or endorsed by the publisher.

## Footnotes

1
http://www.ebi.ac.uk/blastall/nucleotide.html
