# Supplementary material for: Microbiological and Chemical Characteristics of Wet Coffee Fermentation Inoculated With Hansinaspora uvarum and Pichia kudriavzevii and Their Impact on Coffee Sensory Quality
Source: Front Microbiol. 2021 Aug 4;12:713969. doi: 10.3389/fmicb.2021.713969 (PMC8371688; doi:10.3389/fmicb.2021.713969)
Supplement: Supplementary file 1 [file Table_1.pdf]

Supplementary Table 1

Changes in the volatile concentrations in the green and roasted coffee beans from spontaneous (control) and inoculated fermentations with *H. uvarum*, *P. kudriavzevii* and the two yeasts comined.

Volatiles	Green beans					Roasted beans				
	*C**	*Y1*	*Y2*	*Ym*	*P*	*C*	*Y1*	*Y2*	*Y3*	*P*
Acids										
Acetic acid	383.13	547.38	726.84	756.85	0.01	225.90	237.23	262.41	300.52	0.02
Butanoic acid	20.10	12.92	9.73	13.81	0.03	2.85	1.41	2.01	1.78	0.01
Total acids	403.23	560.28	736.54	770.65	0.03	228.75	238.61	264.41	302.28	0.04
Alcohols										
Ethanol	481.72	1573.40	1929.45	1579.95	<.001	5.20	30.42	30.70	37.61	<.001
2-Methyl-3-buten-2-ol	ND	ND	ND	ND		9.38	9.54	10.24	8.71	0.69

Isopentyl alcohol	385.32	446.20	395.62	355.41	0.217	64.80	68.20	59.17	54.01	0.34
Isoamyl alcohol	55.81	183.40	306.12	301.12	<.001	1.40	3.19	2.26	2.66	<.00
3-Methyl-2-buten-1-ol	135.24	127.90	122.10	141.63	0.12	9.38	9.54	10.24	8.71	0.69
3-Hexanol	35.94	33.72	39.4	41.60	0.47	12.30	7.69	9.19	8.46	0.23
2-Nonanol	3.91	4.20	3.81	3.43	0.58	3.10	3.80	2.17	2.93	0.21
2,3-Butanediol	ND	ND	ND	ND		41.93	37.52	39.88	44.27	0.37
Furfuryl alcohol	1.61	1.81	2.23	1.58	0.24	115.50	145.04	146.53	203.67	0.01
1-Nonanol	38.13	27.60	42.12	33.80	0.38	289.32	310.63	381.21	332.52	0.70
2,3-Hexanediol	ND	ND	ND	ND		49.33	59.63	46.30	44.72	0.12
Phenylethyl alcohol	28.78	60.28	110.38	147.09	<.00	2.04	20.34	16.63	13.46	<0.00

1,4-Butanediol	ND	ND	ND	ND		4.80	5.27	4.66	3.98	0.21
Total alcohols	1166.46	2458.51	2951.23	2605.61	0.03	608.48	710.81	759.18	765.71	0.02
Aldehydes										
Acetaldehyde	25.39	37.05	77.47	53.9	<.000	29.70	32.82	48.73	62.44	0.02
3-Methyl butanal	1.83	1.10	2.56	1.41	0.12	53.03	76.74	125.08	246.06	0.04
3-Methyl-2-butenal	57.10	61.31	57.93	49.82	0.32	9.38	9.54	10.24	8.71	0.69
Hexanal	9.56	12.79	16.67	15.31	0.01	9.94	11.74	13.78	12.55	0.01
(*E*)-2-hexenal	42.10	38.12	33.70	34.83	0.34	ND	ND	ND	ND	
2,2-Dimethyl hexanal	110.23	128.05	89.12	113.42	0.68	48.32	39.80	61.27	45.68	0.35
Furfural	ND	ND	ND	ND		1412.06	1416.14	1504.35	1597.08	0.54

Benzaldehyde	10.09	29.70	16.02	14.36	0.033	ND	ND	ND	ND	
5-Methyl furfural	ND	ND	ND	ND		353.06	365.13	342.71	437.22	0.28
Total aldehydes	256.30	308.12	293.47	283.05	0.03	1915.49	1951.91	2106.16	2409.74	0.04
Esters										
Methyl formate	2.6**0**	3.73	5.59	14.42	<.00	21.1**3**	27.4**1**	34.9**4**	50.2**0**	0.04
Methyl acetate	359.62	1727.64	626.7**0**	1345.07	0.02	119**0.2**	164**3.1**	205.88	294.03	0.00
Ethyl acetate	402.97	618.67	334.96	694.34	0.02	9.80	16.52	10.41	20.50	0.02
Methyl butanoate	12.41	5.78	9.35	7.96	<.00	10.16	9.44	8.39	7.91	0.07
Furfuryl formate	ND	ND	ND	ND		4.34	6.65	7.77	11.89	0.00
Furfuryl acetate	ND	ND	ND	ND		62.22	75.40	90.43	138.10	0.002

Furfuryl propionate	ND	ND	ND	ND		77.31	89.60	73.87	81.71	0.91
Total esters	777.60	2351.82	976.60	2061.79	0.02	303.98	389.33	431.69	604.34	0.03
Furans										
2-Methylfuran	ND	ND	ND	ND		10.25	14.15	20.99	32.64	<.00
2,5-Dimethylfuran	ND	ND	ND	ND		10.80	15.91	25.52	45.20	<.00
2-Vinylfuran	ND	ND	ND	ND		29.93	28.12	29.60	32.51	0.87
2-Butyl furan	ND	ND	ND	ND		32.12	28.60	37.31	34.63	0.32
2-Allylfuran	ND	ND	ND	ND		6.23	6.22	9.62	14.42	0.04
2-Acetylfuran	ND	ND	ND	ND		69.52	89.30	106.09	148.43	0.00
2-Propionylfuran	ND	ND	ND	ND		52.62	44.62	33.91	33.57	0.07

2-Furfurylfuran	ND	ND	ND	ND		9.81	8.50	12.72	10.97	0.36
2(5H)-Furanone	ND	ND	ND	ND		2.73	3.62	3.44	4.12	0.25
Tetrahydro-2-(methoxymethyl)furan	ND	ND	ND	ND		1.10	1.20	1.78	3.45	<.00
Total furans	ND	ND	ND	ND		225.11	240.24	280.98	359.94	0.03
Ketones										
2-Butanone	3.62	6.68	4.03	41.67	0.20	15.50	22.92	24.17	33.23	0.02
3-Pentanone	30.78	36.11	46.36	45.17	<.00	0.97	1.19	1.06	1.90	0.02
2,3-Butanedione	4.62	6.20	8.41	12.43	<.00	186.09	275.17	451.36	895.08	0.01
2,3-Pentanedione	ND	ND	ND	ND		326.19	377.33	600.87	1265.33	0.02
2,3-Hexanedione	ND	ND	ND	ND		17.60	17.52	30.84	65.30	0.01

2,3-Heptanedione	ND	ND	ND	ND		1.83	1.90	1.72	1.52	0.24
Total ketones	39.02	48.99	58.80	99.27	0.02	548.18	696.03	1110.02	2262.36	0.03
N-heterocycle										
1-Methylprrole	ND	ND	ND	ND		2.01	2.35	3.17	6.58	0.01
2-Ethylpyrrole	ND	ND	ND	ND		ND	5.20	16.62	4.10	0.04
Pyridine	ND	ND	ND	ND		326.06	386.21	433.88	756.04	<.00
2-Methylpyridine	ND	ND	ND	ND		2.66	3.65	4.36	5.28	0.05
2-Acetylpyridine	ND	ND	ND	ND		6.07	5.45	5.68	7.08	0.27
2-Formyl-1-methylpyrrole	ND	ND	ND	ND		30.92	33.80	30.94	37.31	0.42
4(H)-Pyridine *N*-acetyl-	ND	ND	ND	ND		14.27	14.88	15.91	19.39	0.20

2-Formylpyrrole	ND	ND	ND	ND		19.67	19.65	13.92	10.73	0.00
Total N-heterocycles	ND	ND	ND	ND		401.66	471.19	524.48	846.51	0.02
Phenols										
2-Methoxy phenol (Guaiacol)	ND	ND	ND	ND		3.61	3.18	2.48	2.67	0.29
Phenol	51.20	48.12	49.78	55.29	0.39	59.62	56.20	66.32	71.58	0.28
2-Methyl phenol	ND	ND	ND	ND		63.40	57.94	51.22	52.31	0.12
2-Methoxy-4-vinylphenol	ND	ND	ND	ND		4.29	5.47	5.6	7.26	0.19
Total phenols	51.2	48.12	49.78	55.29	0.43	130.92	122.79	125.62	133.82	0.68
Pyrazines										
Pyrazine	ND	ND	ND	ND		0.58	0.24	1.41	1.43	0.23

2-Methylpyrazine	ND	ND	ND	ND	771.09	871.21	1196.07	1746.32	<.00
2,5-Dimethyprazine	ND	ND	ND	ND	288.25	308.82	441.33	537.11	0.00
2,6-Dimethylpyrazine	ND	ND	ND	ND	115.90	119.72	191.13	238.74	0.003
2-Ethylpyrazine	ND	ND	ND	ND	15.12	17.32	25.16	30.87	0.00
2,3-Dimethylpyrazine	ND	ND	ND	ND	98.52	106.81	134.90	174.18	0.00
2-Ethyl-6-methylpyrazine	ND	ND	ND	ND	96.83	103.81	139.20	165.55	0.05
2-Ethyl-5-methylpyrazine	ND	ND	ND	ND	179.22	168.74	230.12	276.33	0.10
2-Ethyl-3-methylpyrazine	ND	ND	ND	ND	72.32	59.41	75.60	92.67	0.26
3-Ethyl-2,5-dimethylpyrazine	ND	ND	ND	ND	21.64	20.73	24.52	29.51	0.13

2-Acetylpyrazine	ND	ND	ND	ND	5.12	4.73	4.20	3.92	0.51
2-Acetyl-3-methylpyrazine	ND	ND	ND	ND	19.91	18.73	22.80	21.30	0.25
2-Methyl-5-(1-propenyl)pyrazine	ND	ND	ND	ND	7.11	6.70	5.82	6.99	0.55
Total pyrazines	ND	ND	ND	ND	1691.61	1806.97	2492.26	3324.92	0.04
Sulfurs									
Methanethiol	ND	ND	ND	ND	ND	15.71	16.80	13.83	0.32
Dimethyl sulfide	ND	ND	ND	ND	9.13	15.50	28.20	39.72	0.10
Dimethyl disulfide	ND	ND	ND	ND	18.16	17.29	18.81	15.90	0.12
Dimethyl trisulfide	ND	ND	ND	ND	39.11	26.20	29.93	33.41	0.41
Furfuryl methyl sulfide	ND	ND	ND	ND	22.93	18.60	33.88	23.31	0.24

Bis-2-(furfuryl)-disulfide	ND	ND	ND	ND	8.62	5.50	8.12	6.76	0.23
Total sulfurs	ND	ND	ND	ND	97.95	98.80	135.74	132.93	0.47

The data are means of three replicates. Abbreviations**:** C, spontaneous fermentation (control); Y1, *Hansinaspora uvarum* inoculation; Y2, *Pichia kudriavzevii* inoculation;Ym combined yeasts inoculation, p, *p*-Value, ND, not detected.
